# An Autonomous Thermal Camera System for Monitoring Fumarole Activity

**DOI:** 10.3390/s24061999

**Published:** 2024-03-21

**Authors:** Harald van der Werff, Eunice Bonyo, Christoph Hecker

**Affiliations:** 1 Faculty for Geo-Information Science and Earth Observation, University of Twente, 7500 AE Enschede, The Netherlands; c.a.hecker@utwente.nl; 2 Kenya Electricity Generating Company PLC (KenGen), Nairobi P.O. Box 47936, Kenya; ebonyo@kengen.co.ke

**Keywords:** autonomous, camera, thermal infrared, time-series, fumarole, geothermal, Olkaria

## Abstract

The Kenyan part of the East African Rift System hosts several geothermal fields for energy production. Changes in the extraction rate of geothermal fluids and the amount of water re-injected into the system affect reservoir pressure and production capacity over time. Understanding the balance of production, natural processes and the response of the geothermal system requires long-term monitoring. The presence of a geothermal system at depth is often accompanied by surface manifestations, such as hot water springs and fumaroles, which have the potential for monitoring subsurface activity. Two thermal camera timelapse systems were developed and installed as part of a multi-sensor observatory in Kenya to capture fumarole activity over time. These cameras are an aggregation of a camera unit, a control unit, and a battery charged by a solar panel, and they monitor fumarole activity on an hourly basis, with a deep sleep of the system in between recordings. The article describes the choice of hardware and software, presents the data that the cameras acquire, and discusses the system’s performance and possible improvement points.

## 1. Introduction

**Figure 1:**
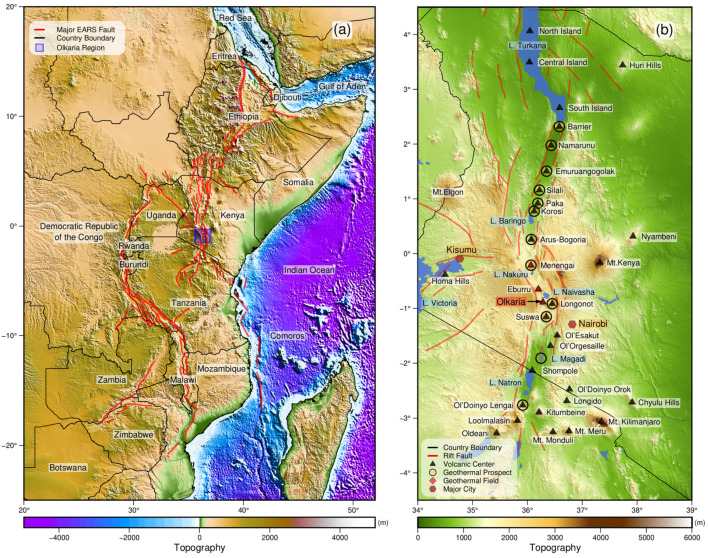
(**a**) The East African Rift System (EARS) with continental surface expressions indicated in red. The Kenya Rift System is the part of the EARS that crosses Kenya. (**b**) Volcanoes and geothermal fields along the Kenya Rift System. The figure is re-used without modification and with permission from Fadel et al. [5] following the https://creativecommons.org/licenses/by-nc-nd/3.0/us/ CC BY-NC-ND 3.0 license (accessed on 15 March 2024).

Heat from volcanic geothermal systems is a sustainable energy source with low carbon dioxide emissions. Hot fluids and steam contained in a subsurface reservoir are brought to the surface and used to produce electricity [1,2,3]. The most substantial geothermal resources are usually found near active tectonic plate boundaries or volcanic regions [4]. The East African Rift System is one of the most promising prospects for geothermal energy [5]. This system extends from the Red Sea and the Gulf of Aden to Mozambique and Botswana and hosts several geothermal active areas (Figure 1). Kenya commissioned the first geothermal plant in 1981 and two more units in 1982 and 1983. In 2019, the country ranked eighth globally in terms of geothermal energy utilization with an installed capacity of 865 MW [6]. Currently, it ranks seventh with 944 MW installed. Most geothermal energy production in Kenya comes from a single field, the Olkaria geothermal field. The Kenya Electricity Generating Company PLC (KenGen) produces roughly 799 MW in this field, which is 81% of the field’s production and 38% of the total energy production in Kenya.

Olkaria is a high-temperature geothermal system characterized by a magmatic heat source, a porous reservoir of volcanic rocks, and a clay cap of hydrothermally altered rocks that overlie the reservoir and keep the hot fluids contained. Large-scale convection within the reservoir circulates fluids upward in up-flow zones, whereas cooled fluids and meteoric water move downward to recharge the reservoir. This convection carries heat from the deep subsurface to shallower levels, where it can be extracted through geothermal production wells [5]. Changes in the extraction rate of geothermal fluids, and possibly the amount of water available to the system due to precipitation, could cause the geothermal motor to run at different speeds. Despite liquids being re-injected back into the reservoir after energy generation, the overall mass of extraction exceeds what is re-injected; in the long term, balancing is needed to maintain reservoir pressure and production capability. Finding this balance requires long-term monitoring of production, natural processes, and the response of the geothermal system.

A geothermal system often has surface manifestations such as hot water springs, steaming grounds, fumaroles, and hot grounds [1]. In Olkaria, numerous volcanic craters, old lava flows, hot grounds, and fumaroles can be found. The fumaroles exhibit temperatures of over 363 K (90 °C). The geothermal activity and associated heat fluxes measured at the surface can vary over thousands of years [7], decades [8], and even seasonally [9]. To capture some of these natural fluctuations in fumarole activity, as well as those related to the operation of the reservoir for electricity production, long-term monitoring solutions are required to operate autonomously for extended periods with low maintenance pressure. Since the optimal observation time-of-day for monitoring these fumaroles was not known, time-series recordings with an observational frequency of once per hour were chosen as the trade-off between data availability, data volume, and power consumption.

This article describes two thermal-infrared time-lapse cameras that monitor fumaroles in Olkaria to capture, as part of a multi-sensor field observatory, the geothermal system’s dynamics. In particular, the article describes the choice of hardware, the control software, and the data that the cameras provide. We compare the camera results with data from a nearby weather station and a climate reanalysis dataset and discuss the system’s performance so far and possible improvements.

## 2. The Thermal Camera System

### 2.1. Hardware

The design of the cameras has several commonalities with the equipment presented by Peters et al. [10], who uses an autonomous thermal infrared camera for continuous monitoring of an active lava lake. In Olkaria, these cameras monitor fumarole activity hourly, with a deep sleep of the system in between recordings. This led to different choices for hardware and software solutions, which are presented below.

**Figure 2:**
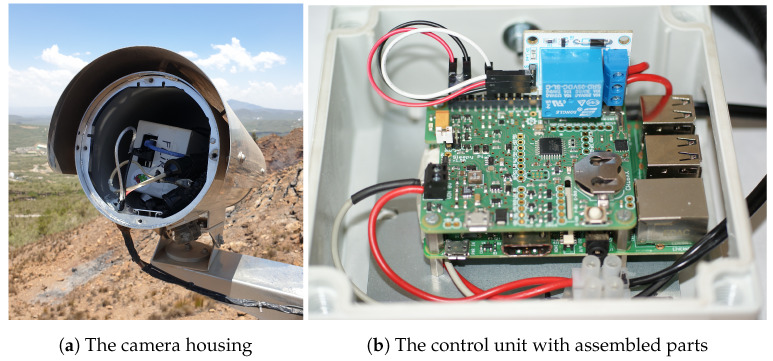
(**a**) The opened camera housing in the field and (**b**) the opened control unit.

The camera system is an aggregation of a thermal camera unit and a control unit placed in an air-tight stainless steel enclosure (Figure 2a) and a 12 V battery charged by a solar panel in a separate housing. Except for the control unit, all components are off-the-shelf parts that only need connecting. The control unit itself consists of several off-the-shelf parts that need assembling (Figure 2b) and require a custom code to read and process camera data. The camera, control unit and customized code are discussed in detail in the following sections.

The camera unit is an FLIR A655SC, which has an uncooled microbolometer that measures in a 7.5 μm to 14 μm wavelength range [11]. The factory calibration of the camera is saved as non-uniformity compensation (NUC) coefficients which are applied during operation to maintain image quality. The camera automatically selects the optimum calibration table based on its temperature [12]. We compensate for possible drift of the camera by triggering an NUC before data acquisition.

The power for the camera unit comes via a 12 V DC connector which, when enabled via a switched relay in the control unit, directly runs from the 12 V battery. The non-proprietary GenICam interface [13] allows capture parameters to be set and recorded frames to be received via a 1 Gbit Ethernet connection. In our system, we set several capture parameters via the control unit: 640 × 480 pixels at a 3 Hz single frame acquisition mode, a 10 mK linear temperature in 16 bit integer format, and focus distance by proxy of a digital number for the focus ring position on the lens. The lens position is, for each location where the camera is installed, found in the field by averaging 3 autofocus repeats with the manufacturer’s software [14], and the focus ring position is read out via GenICam interface before running the camera autonomously. During autonomous image capture, the implied focus distance is read back from the camera and written into a log file (together with other environmental parameters, detailed below). Found by experimenting, we decided to capture, for each acquisition, about 30 frames and average these to reduce noise and avoid occasional frame dropouts.

The control unit is based on a single-board computer that is connected to the camera via the Ethernet. We use a “Raspberry Pi 3 Model B” with a quad-core 1.2 GHz ARM CPU, 1 Gbit RAM, and a 100-Base Ethernet adapter [15]. Although this board does not have the 1 Gbit Ethernet adapter that is according to the manual recommended for this camera, this setup appears to work as long as certain precautions are taken (discussed in the Deployment section). The onboard Bluetooth and WiFi adapter are disabled by the operating system to save power. The Raspberry Pi only wakes up to take the hourly measurement.

Always on is a “Sleepy-Pi 2” add-on board, which connects directly to the Raspberry Pi [16]. This board is equipped with an Arduino microcontroller and a real-time clock with a backup battery. It controls the power to the Raspberry Pi with a 5 V 10 A switched relay, which allows the Raspberry Pi to enter a deep sleep mode, wake up at a specific time, or act when an event is triggered (e.g., when a certain amount of time has passed). The Sleepy-Pi also controls a relay that switches power to the camera. The Sleepy-Pi accepts 5 V to 30 V DC power input and can therefore run, together with the Raspberry Pi, from the same 12 V DC power source as the FLIR camera. The Raspberry Pi reportedly needs a current of approx. 0.5 A during normal operation, making the total power consumption approx. 2.5 W [17]. With the Raspberry Pi powered down, the Sleepy-Pi alone uses approx. 180 mA, dropping the total power consumption to approx. 0.9 W.

Although accurate time stamping is not essential for our measurements, hardware clocks could drift over time. Consequently, the control unit is equipped with a “GlobalSat G-Star IV USB GPS” receiver [18] to keep the clock synchronized. The Raspberry Pi obtains its system time from the Sleepy-Pi when it boots. Directly after booting, the “network time protocol daemon” [19] is started to obtain GPS time. Depending on the satellite constellation of that moment, it may take several minutes for the GPS to obtain a lock and for the system time to become synchronized. Only when the system time is synchronized with the GPS, the system time is written to the real-time clock on the Sleepy-Pi. This means that time synchronization is in effect after a measurement has been taken and comes into effect at the next event.

Several sensors on the camera, the Raspberry Pi, and the Sleepy-Pi are being logged. The camera can report its settings and several environmental parameters via the GenICam interface; this information is written to a text file with every image that is saved to disk. Specifically, the focus distance of the camera and two of the six internal temperatures are monitored: The temperatures reported on the front, lens, and shutter appear to have similar readings all of the time, and the same holds for temperatures reported for the atmosphere, external optics, and reflected temperature. In addition, the Raspberry Pi reports the temperature of the CPU and the time reported by the hardware clock, the operating system, and the GPS. The Sleepy-Pi reports on the input voltage coming from the battery.

As the control unit is not equipped with telemetry, the camera measurements and sensor readings are primarily stored on the flash memory card in the Raspberry Pi, which also hosts the operating system. To reduce the chances of data loss and simplify regular data readout, the data on the flash memory card are also synchronized to a USB stick which is accessible outside of the camera enclosure. As synchronizing the image collection to an empty USB stick takes time, this task is carried out immediately in the 5 min after boot when the system is waiting for the camera to warm up and stabilize. After taking a measurement, the newest results are also added to the USB stick before the Raspberry Pi goes back to sleep.

**Figure 3:**
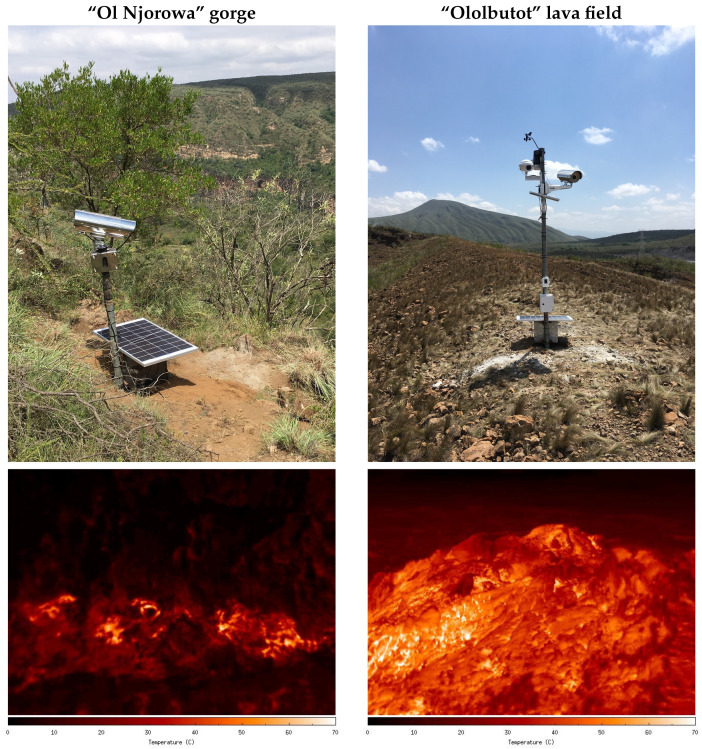
The two observation towers deployed in Olkaria, with a photo of the hardware deployed in the field (**top**) and the thermal IR image recorded by the camera (**bottom**). On the left is the installation at the “Ol Njorowa” gorge, consisting of a thermal camera only. On the right is the installation at the “Ololbutot” lava field, consisting of a thermal camera, a timelapse camera, and a meteostation.

The camera and the control unit are placed in a weather-proof and air-tight enclosure made of stainless steel, which should be resistant to acidic gases coming out of fumaroles. The enclosure is a customized “Tecnovideo 204SHIR70” with a 7 cm diameter Germanium window, an added sun shield on top, and no heater nor window wiper. The enclosure is mounted on a steel pole. The 12 V 65 A h battery and solar charger are placed in a separate custom-made metal enclosure on the ground, which is covered by a 100 W solar panel (Figure 3). The power cables running from the battery to the control unit are uninterrupted, and “Terostat-IX” putty is used to seal the cable openings in the enclosure. Silica gel packages keep the airtight enclosure dry; these need replacing when the enclosure is opened for a longer time during maintenance.

### 2.2. Software

The Sleepy-Pi has a custom code created with Arduino IDE 1.0.5 [20] that reads the battery voltage and boots the Raspberry Pi at hourly intervals. The Raspberry Pi has a GNU/Linux operating system (Raspbian 9.8, codename “Stretch” [21]). An initialisation script written in Bash [22] is launched automatically when booting the Raspberry Pi. This script calls other shell scripts that are wrappers around programs needed to bring up the network connection to the camera, operate the camera (upload settings, trigger a NUC, capture and download an image), read the GPS and synchronize time, read the multiple sensors on the camera and in the control unit, perform data management, and perform a controlled shutdown after measurements have been taken. The camera itself is operated with the “Aravis” library [23] that allows communication with the camera over the GenICam interface. A custom-made Python [24] script is used to set camera parameters and receive the captured frames. A link to the developed scripts is in the “Data Availability Statement” below.

## 3. Deployment

Two thermal camera systems are deployed in Olkaria; one is overlooking a fumarole in the “Ol Njorowa” gorge, and the other is looking at a fumarole in the “Ololbutot” lava field (Figure 3). At the gorge, the camera system is positioned at the edge of the escarpment. A 41 mm telephoto lens is used to observe the fumarole at the valley floor at a distance of about 500 m. A 13 mm wide-angle lens is employed at the lava field to image the whole fumarole at distances from 15 m to 30 m. Instruments installed alongside the thermal camera system are a “Cyclapse” autonomous time-lapse camera system [25] to observe ground motion at the fumarole, and a Davis “Vantage Pro 2” meteostation [26] to aid the interpretation of the thermal camera data. Both systems were installed in July 2019, received their first maintenance (with a software update for improved logging of system parameters) in July 2020 and were serviced again in February 2022. KenGen staff do a monthly check-up of the system and exchange USB sticks to retrieve data.

### 3.1. “Ol Njorowa” Gorge

**Figure 4:**
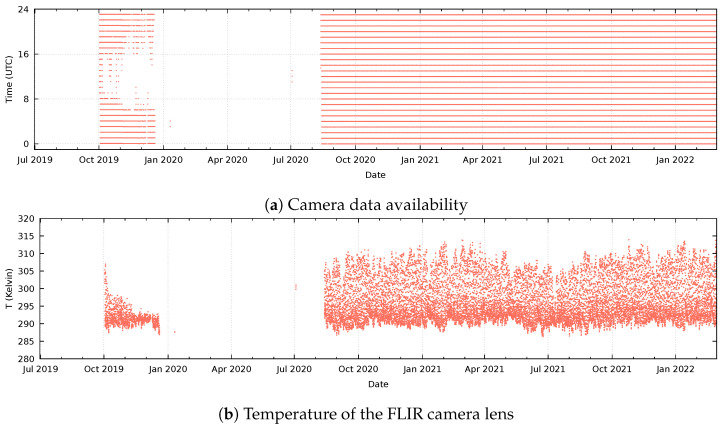

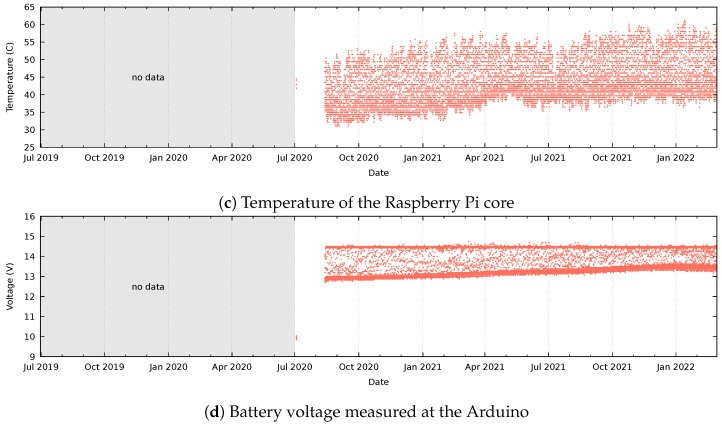
Camera system parameters at the “Ol Njorowa” gorge. From top to bottom, the records of (**a**) data availability and (**b**) camera lens temperature, available since the installation of the camera in July 2019, and the records of (**c**) core temperature of the Raspberry Pi in the control unit and (**d**) the battery voltage at the Arduino, measured as of July 2020.

Figure 4 shows data availability and camera parameters recorded at the “Ol Njorowa” gorge. This camera initially had problems starting up. After a checkup by KenGen staff, it worked for a short period but then failed again in December 2019. Downtime and resulting data loss appeared to be caused by a failing power supply: During maintenance in July 2020, it appeared that the solar panel was erroneously connected to the charge controller; this explains an initial loss of data during daylight hours and the eventual failure due to battery exhaustion. The system resumed functioning after the battery was also replaced in August 2020. The average temperature of the thermal camera varies between 288 K and 314 K without a clear seasonal change visible in the data (Figure 4b).

The readings in the control unit started after updating the control unit in July 2020. The temperature readings of the Raspberry Pi (Figure 4c) match the fluctuations of the camera temperature precisely (Figure 4b). There is however a difference in accuracy, as the Raspberry Pi reading shows a gradual increase over time that is not reflected in the camera temperature. The battery status (Figure 4d) shows that the lower (night) voltage level increased over time, reflecting an improvement in the battery condition, until it seems to stabilize in January 2022.

### 3.2. “Ololbutot” Lava Field

**Figure 5:**
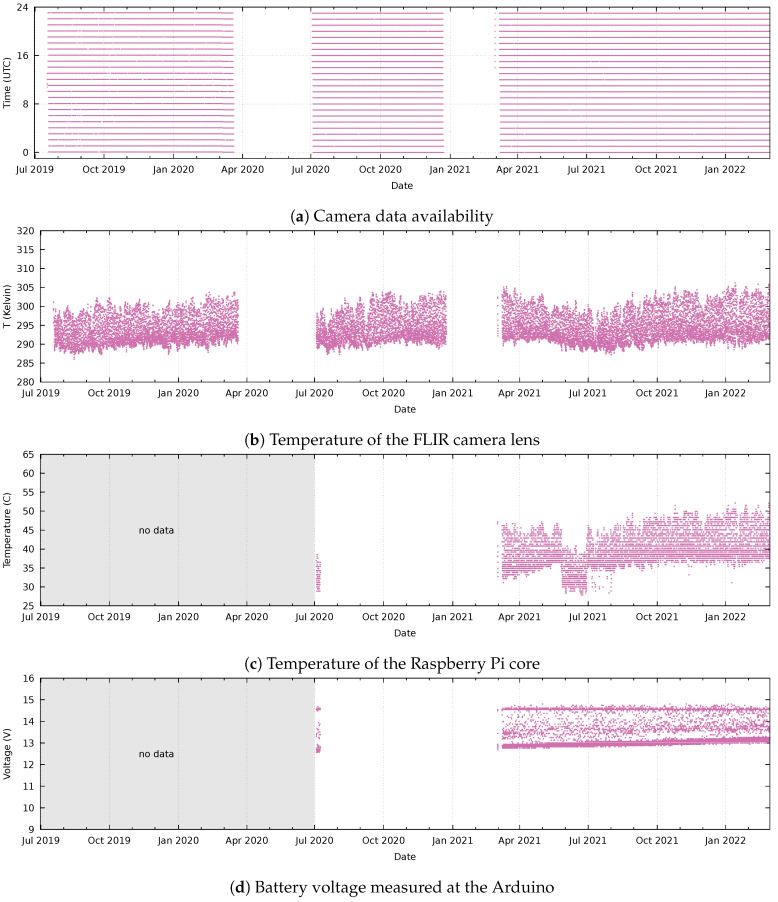
Camera system parameters at the “Ololbutot” lava field. From top to bottom, the records of (**a**) data availability and (**b**) camera lens temperature, available since the installation of the camera in July 2019, and the records of (**c**) core temperature of the Raspberry Pi in the control unit and (**d**) the battery voltage at the Arduino, measured as of July 2020.

Figure 5 shows data availability and camera parameters recorded at the “Ololbutot” lava field. After the installation, the camera recorded hourly data until March 2020. When the enclosure was opened during maintenance in July 2020, it was found that some power cables within the enclosure had come loose. The cause for this unforeseen damage has not been found. After replacing damaged cables and the control unit, the system functioned until the end of December and resumed working again in February 2021 after replacing a failed battery. The temperature recorded by a sensor at the camera lens varies between 288 K and 305 K (Figure 5b). A difference with the camera at the gorge is that the upper temperature is on average ±10 K lower, and there seems to be a seasonal variation.

The readings in the control unit started after replacing the control unit in July 2020. For unknown reasons, the logging failed soon after despite the camera continuing to function; the readings resumed in March 2021. The temperature of the Raspberry Pi (Figure 5c) seems precise as the smaller fluctuations match that of the camera temperature (Figure 5b). There is a difference in accuracy, where the Raspberry Pi temperature reading gradually increases over time. Furthermore, in June 2021, there was an unexplained deviation in the Raspberry Pi temperature readings which is not reflected in the camera temperature readings. The battery status shows that the lower voltage limit increased over time (Figure 5d), reflecting a continuous improvement in the battery condition.

During the 2022 maintenance, the solar panel appeared slightly corroded. This may be a result of aggressive exhalations coming from the nearby fumarole. However, due to the overcapacity of the panel, there is no concern about the power status in the short run, and the battery voltage remains excellent (Figure 5d). The germanium window of the enclosure did not show any signs of precipitates. The inside of the housing was clean and no corrosion was visible, suggesting that the airtight seal made of putty works as expected.

### 3.3. Comparison with Other Sources

**Figure 6:**
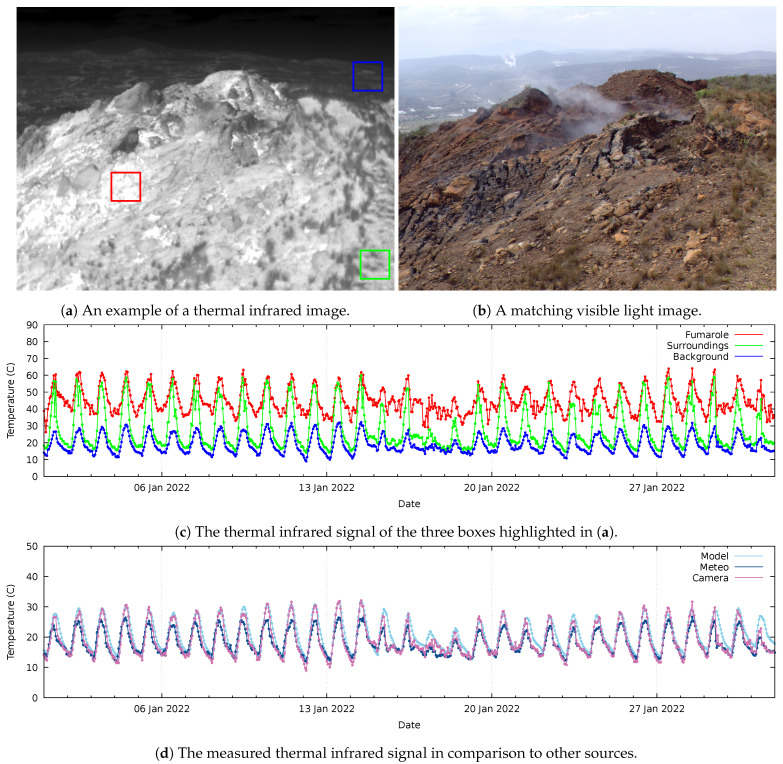
The observations made at the “Ololbutot” lava field. (**a**) shows the complete view of the thermal IR camera (bright equals warm); (**b**) shows an image of the SLR time-lapse camera covering the same area. The coloured regions in (**a**) indicate the area of pixels sampled for fumarole (•) values, the direct surroundings with bits of vegetation (•), and background temperatures in the distance (•). (**c**) shows the average values of the sampled pixels in the three boxes over January 2022. (**d**) shows a comparison of the thermal infrared camera records against other data sources over time. The camera data (•) are compared to the air temperature measured by the meteostation (•) installed at the same location, as well as the topsoil (•) temperature of the ERA5-Land reanalysis model.

Figure 6 illustrates the data acquired by the camera at the “Ololbutot” lava field. Figure 6a shows an example of a recorded thermal infrared image, while Figure 6b shows a corresponding image of the time-lapse camera. This geothermal surface manifestation is characterized by a fumarole that vents hot gases and steam through multiple cracks in the ground surface, which can be seen in Figure 6b. The red box in the thermal infrared image in Figure 6a reveals in bright tones that the soil surrounding these cracks is relatively warm. The area in the background, indicated by the blue box, is not part of the lava field and appears cooler with darker tones in the thermal image. The soil with patches of vegetation, indicated by the green box to the right of the fumarole and in the foreground, is cooler than the fumarole itself.

Figure 6c shows a month of temperature readings, sampled from the ground with cracks (red box), the direct surroundings of the fumarole (green box), and the distant background outside of the lava field (blue box). This figure shows that the temperature in the fumarole is relatively high and that the temperature of the background is relatively low. Fumarole temperatures also do not typically drop below 30 °C at night. The direct surroundings of the fumarole (green box) reach the high temperatures of the fumarole during the day but come close to the background temperature at night.

Figure 6d compares the camera data with air temperature readings of the Davis meteostation mounted at the same location, at 4 m above the soil surface. These time series are also compared to temperature data taken from the ERA5-Land reanalysis model of the European Centre for Medium-Range Weather Forecasts (ECMWF) [27], which comes at an approximately 11 km spatial resolution. The model parameter used is “soil_temperature_level_1”, which is the soil temperature at 0 cm to 7 cm depth. All three datasets highlight the weather event that took place from 15 to 19 January.

## 4. Discussion

From looking at the observations in Figure 6, the daily temperature cycles show a high positive correlation. It can be concluded that the radiant temperature recordings of the camera and air temperature of the meteostation correspond with the modelled top-soil temperature of the ERA5-Land re-analysis model. However, within each daily cycle, there are deviations of multiple degrees possible.

The combination of a Raspberry Pi with an Arduino microcontroller is suited for automated observations at specific times of the day or regular intervals: The system sleeps and wakes without problems and comes with low power consumption. The data are momentarily stored on an SD card inside the control unit and on a USB stick outside of the enclosure. The USB sticks can be exchanged for data collection and data are synchronized with those on the internal SD card. This design ensured that data could be read out at any time and did not lead to data loss. However, with growing data volume, not only the time needed to copy data increases but also the time needed to compare between the SD card and the USB stick increased in the synchronization process. We experienced that after a year of data collection (±8 GB), 5 min were insufficient to finish the data comparison, let alone copy the data. In the software update carried out in July 2020, the synchronization process was, therefore, replaced with a simple copy statement only involving the last recorded data.

In general, the battery condition improved over time, indicating that the design helps to save power in between measurements. In addition, we can conclude that the setup with a marine battery charged by a 100 W solar panel is sufficient for long-term operation in geographic areas close to the equator. However, the Achilles heel of these camera systems is the power supply. Apart from the panel connection error made while setting up the camera system at the gorge, the longer-term data outages could be related to sudden battery breakdowns. A lack of telemetry installed with the system did not allow any early warnings to be sent.

From practical experience in the field, several possible improvements to the design have come up:

1. The Raspberry Pi computer in the control unit should be accessible remotely or be made hot-swappable in the field to be able to troubleshoot problems.
2. There should be no cables going through the side of the housing. Instead, these should be interrupted by sealed bayonet connectors. Apart from the severed cables that we encountered inside one of the camera units, USB connectors could loosen over time by pulling cables on the outside.
3. If possible, all camera data, but at least the system health data, should be regularly sent by a wireless connection. This could for example warn of a degrading battery.

The camera data will be used in the long-term monitoring of fumaroles as surface manifestations of a geothermal system underground. A current debate is whether extracting fluids in geothermal exploitation leads to a decrease in activity in surface manifestations due to fluid extraction, or that dropping pressure leads to increased generation of steam, thereby increasing surface manifestation activity. We plan to investigate this problem with the thermal images provided by our camera units.

## 5. Conclusions

This article introduced the design of two timelapse camera systems equipped with a FLIR A655SC thermal bolometer camera and controlled by a Raspberry Pi single-board computer and an Arduino microcontroller. The design resembles an existing design made for continuous monitoring [10] but was adapted to be able to enter deep sleep in between hourly recordings.

The daily temperature cycles recorded by the cameras showed a positive correlation with top-soil temperature data obtained from the ERA5-Land re-analysis model and air temperature of a meteostation mounted close to the camera. Furthermore, the camera data correlated with temperature data read from the control computer, being more accurate than precise.

The purpose of the two deployed cameras was to monitor fumarole activity in an actively utilized geothermal system to aid in monitoring the geothermal reservoir’s response to fluid extraction and re-injection cycles, and while the suitability of these data for that specific purpose is yet to be tested, the design of the camera units was found to meet our demands.

## Data Availability

The camera data acquired in this work is under embargo but will be published at a later stage. The code developed for this work is published on https://github.com/haraldvanderwerff/flir-timelapse-controller, (accessed on 15 March 2024).

## Abbreviations

The following abbreviations are used in this manuscript:

ARM	Advanced RISC Machines
CPU	Central Processing Unit
DC	Direct Current
ECMWF	European Centre for Medium-Range Weather Forecasts
ERA5	ECMWF ReAnalysis v5
FLIR	Forward-looking infrared
GNU	GNU’s Not Unix!
GPS	Global Positioning System
LWIR	Long-wave InfraRed
PLC	Public Limited Company
RAM	Random Access Memory
RISC	Reduced Instruction Set Computer
SD	Secure Digital
USB	Universal Serial Bus

## References

[1] van der Meer F., Hecker C., van Ruitenbeek F., van der Werff H., de Wijkerslooth C., Wechsler C. (2014). Geologic remote sensing for geothermal exploration: A review. Int. J. Appl. Earth Obs. Geoinf..

[2] Lee K.C., Cleveland C.J. (2004). Geothermal Power Generation. Encyclopedia of Energy.

[3] Dickson M.H., Fanelli M. (2003). Geothermal Energy: Utilization and Technology.

[4] Limberger J., Boxem T., Pluymaekers M., Bruhn D., Manzella A., Calcagno P., Beekman F., Cloetingh S., van Wees J.D. (2018). Geothermal energy in deep aquifers: A global assessment of the resource base for direct heat utilization. Renew. Sustain. Energy Rev..

[5] Fadel I., Hecker C., Kimata J., Bonyo E., van der Meijde M., van der Werff H., van der Meer F. (2021). Geoscientific monitoring of Olkaria’s geothermal motor. EOS.

[6] Omenda P., Mangi P., Ofwona C., Mwangi M. Country update for Kenya 2015–2019. Proceedings of the World Geothermal Congress, International Geothermal Association.

[7] Sturchio N.C., Dunkley P.N., Smith M. (1993). Climate-driven variations in geothermal activity in the northern Kenya rift valley. Nature.

[8] Mia M.B., Bromley C.J., Fujimitsu Y. (2012). Monitoring heat flux using Landsat TM/ETM+ thermal infrared data — A case study at Karapiti (‘Craters of the Moon’) thermal area, New Zaland. J. Volcanol. Geotherm. Res..

[9] Ingebritsen S., Galloway D., Colvard E., Sorey M., Mariner R. (2001). Time-variation of hydrothermal discharge at selected sites in the western United States: Implications for monitoring. J. Volcanol. Geotherm. Res..

[10] Peters N., Oppenheimer C., Kyle P. (2014). Autonomous thermal camera system for monitoring the active lava lake at Erebus volcano, Antarctica. Geosci. Instrum. Method. Data Syst.

[11] FLIR A655SC LWIR Camera. https://www.flir.eu/products/a655sc/.

[12] FLIR—A35 & A65 Cameras Manual Control of the NUC. https://flir.custhelp.com/app/answers/detail/a_id/1319/related/1.

[13] The Generic Interface for Cameras Standard. https://www.emva.org/standards-technology/genicam/.

[14] ResearchIR Software. https://www.flir.eu/support/products/researchir#Overview.

[15] Raspberry Pi 3 Model B. https://www.raspberrypi.com/products/raspberry-pi-3-model-b/.

[16] Sleepy Pi 2-Micro USB 2. https://spellfoundry.com/product/sleepy-pi-2/.

[17] Sleepy Pi 2 FAQ. https://spellfoundry.com/docs/sleepy-pi2-faq/#4-toc-title.

[18] Cable GPS with USB Interface (SiRF Star IV). https://www.globalsat.com.tw/en/product-199952/Cable-GPS-with-USB-interface-SiRF-Star-IV-BU-353S4.html.

[19] The Network Time Protocol. https://www.ntp.org/.

[20] The Arduino Integrated Development Environment. https://www.arduino.cc/en/software.

[21] Raspbian 9.8. http://www.raspbian.org/.

[22] The GNU Project’s Bourne Again SHell. https://www.gnu.org/software/bash/.

[23] A Vision Library for Genicam Based Cameras. https://github.com/AravisProject/aravis.

[24] Welcome to Python.org. www.python.org.

[25] Cyclapse Time-Lapse Camera System. https://web.archive.org/web/20230831002228/https://cyclapse.com/.

[26] Vantage Pro 2 Weather Station. https://www.davisinstruments.com/pages/vantage-pro2.

[27] Muñoz Sabater J. (2019). ERA5-Land Hourly Data from 1981 to Present. Copernicus Climate Change Service (C3S) Climate Data Store (CDS). https://cds.climate.copernicus.eu/cdsapp#!/dataset/10.24381/cds.e2161bac?tab=overview.

